# Photoperiodic Regulation of Growth-Dormancy Cycling through Induction of Multiple Bud–Shoot Barriers Preventing Water Transport into the Winter Buds of Norway Spruce

**DOI:** 10.3389/fpls.2017.02109

**Published:** 2017-12-11

**Authors:** YeonKyeong Lee, Chithra Karunakaran, Rachid Lahlali, Xia Liu, Karen K. Tanino, Jorunn E. Olsen

**Affiliations:** ^1^Department of Plant Sciences, Faculty of Biosciences, Norwegian University of Life Sciences, Ås, Norway; ^2^Canadian Light Source Inc., Saskatoon, SK, Canada; ^3^Department of Plant Sciences, College of Agriculture and Bioresources, University of Saskatchewan, Saskatoon, SK, Canada

**Keywords:** cell wall modification, crown structure, dormancy, Norway spruce, methyl-esterification status of homogalacturonan pectin, plasmodesmata blocking, bud–shoot barrier, xylem blockage

## Abstract

Whereas long days (LDs) sustain shoot elongation, short days (SDs) induce growth cessation and formation of dormant buds in young individuals of a wide range of temperate and boreal tree species. In specific conifers, including Norway spruce, photoperiodic control of bud development is associated with the formation of a plate of thick-walled cells, denoted as the crown, at the base of the bud. Information about cellular characteristics of this crown region is limited. We aimed to test whether the crown region is an important SD-induced barrier ensuring dehydration of the developing winter bud by preventing water influx. Using microscopy and synchrotron techniques, we show here that under LD, cell walls in growing shoot tips had highly methyl-esterified homogalacturonan pectin. During SD-induced bud development, the homogalacturonan in the crown region was de-methyl-esterified, enabling Ca^2+^ binding and crosslinking, a process known to decrease cell wall water permeability by reducing pectin pore size. In addition, there was abundant callose deposition at plasmodesmata in the crown region, and xylem connections between the bud and the subtending shoot were blocked. Consistent with reduced water transport across the crown region into the bud, uptake of fluorescein in shoot tips was blocked at the base of the bud under SD. Upon transfer from SD to bud-break-inducing LD, these processes were reversed, and aquaporin transcript levels significantly increased in young stem tissue after 4 weeks under LD. These findings indicate that terminal bud development is associated with reduced water transport through decreased cell wall permeability and blocking of plasmodesmata and xylem connections in the crown structure. This provides further understanding of the regulatory mechanism for growth-dormancy cycling in coniferous tree species such as Norway spruce.

## Introduction

To survive the winter, tree species of the temperate and boreal zone need to cease their growth before the onset of low temperature. The timing of growth cessation is critical and leads to a chain of events resulting in the development of terminal dormant buds, bud dormancy, and cold hardiness (Olsen, 2010). Lang (1987) defined dormancy as a developmental process involving a temporal suspension of growth of any plant structure containing a meristem. Rohde and Bhalerao (2007) proposed to redefine dormancy as the inability to initiate growth from meristems or other organs and cells with the capacity to resume growth under favorable conditions.

In young individuals of a wide range of woody species of the temperate and boreal zone, photoperiod (or rather night length) is the primary factor controlling the growth-dormancy transition. Growth is sustained under long days (LDs; short nights) and growth cessation generally occurs under short days (SDs; long nights) in which the photoperiod is shorter than the critical length sustaining growth (Nitsch, 1957; Jackson, 2009). As an adaptation to the duration of the growing season at different sites, the critical photoperiod for growth increases with increasing northern latitude. Growth-dormancy cycling in tree species includes substantial morphological changes from a growing terminal shoot to a terminal bud with leaf initials enclosed by bud scales. The bud gradually enters deep winter dormancy and attains cold hardiness in response to the shortened photoperiod as well as reduced temperature (Welling and Palva, 2006; Olsen, 2010; Tanino et al., 2010). Subsequently, dormancy is alleviated, and this is followed by de-hardening, bud burst and re-initiation of growth. In a wide range of woody species dormancy release requires a period of low temperature. In Norway spruce, at least in young plants, low temperature hastens dormancy alleviation, but is not strictly needed (Nienstaedt, 1967; Worrall and Mergen, 1967; Søgaard et al., 2008; Lee et al., 2014; Olsen et al., 2014).

The growth-dormancy cycling are accompanied by a multitude of changes in the transcriptome, proteome, and metabolome (Moritz and Olsen, 1995; Ruttink et al., 2007; Olsen, 2010; Asante et al., 2011; El Kayal et al., 2011; Ko et al., 2011; Paul and Kumar, 2011; Lee et al., 2014; Howe et al., 2015). Development of the dormant winter bud and cold hardiness as well as dormancy release, de-hardening and bud burst are well known to be related to changes in the levels of plant hormones (reviewed by Olsen, 2010). Under SD, the levels of gibberellin and abscisic acid (ABA) decrease and increase, respectively, whereas upon bud burst, the situation is reversed. ABA acts a signal in cold hardening, which is associated with dehydration and osmoregulation, among others through the accumulation of sugars to prevent freezing (Welling and Palva, 2006). Furthermore, many woody species exhibit deep supercooling, which make them able to prevent ice crystal formation in their cells by inhibiting ice nucleation at freezing temperatures (Burke et al., 1976; Kang et al., 1998; Wisniewski et al., 2003). The restriction of ice growth by supercooling is promoted by ice barriers such as thick-walled cells containing lignin, suberin, and cutin (Kuprian et al., 2014, 2016, 2017).

In species of the subfamilies *Piceoideae*, *Abietoideae*, and *Lariccoideae* of the pine family (*Pinaceae*), a cell plate commonly denoted as the crown, which consists of thick-walled cells, appears across the pith at the base of the terminal bud during the development of the winter bud (Owens and Molder, 1976; Sakai, 1979; MacDonald and Owens, 1993). The crown structure was suggested to be a physical barrier restricting the transport of water into the terminal bud. Consequently, the crown prevents ice propagation from the shoot axis into the shoot tip (Sakai, 1979; MacDonald and Owens, 1993). While several studies have addressed bud morphology and anatomy in Norway spruce and other *Picea* species under LD and SD (Sakai, 1979; El Kayal et al., 2011; Sutinen et al., 2012; Olsen et al., 2014), specific mechanisms related to structure–function relationships and biochemical changes *in situ* in regulation of winter bud formation are still not well understood.

Plant cell walls are key structural components providing rigidity. Cell walls contain cellulose, hemicelluloses, pectins, lignin, and proteins as major components (Sarkar et al., 2009). The cellulose backbone consists of straight chains of glucose units forming microfibrils. These cellulose microfibrils are linked via hemicellulose tethers to form a cellulose–hemicellulose network embedded in a pectin matrix.

Pectin, which is the most abundant component in the primary cell walls and middle lamella, is a polysaccharide with a high content of 1,4-linked α-D-galactosyluronic acid residues (Ridley et al., 2001). Pectin can be divided into three main groups, the linear homogalacturonan (HG) and the substituted rhamnogalacturonans (RGs) RG-I and RG-II. HG is composed of unbranched homopolymers of (1→4)-α-D-galacturonic acid (GalA) residues with varying degree of methyl-esterification of the carboxyl groups. Plant cell wall HG is synthesized as the methyl-esterified form, which is later modified to the de- or partially methyl-esterified form by pectin methyl esterase (PME, EC 3.1.1.11) enzymes (Knox et al., 1990; Xu et al., 2011). The HG with a low degree of methyl-esterification readily forms an ordered gel structure commonly denoted as an ‘egg-box’ structure in the presence of calcium ions (Ca^2+^) (Grant et al., 1973; Braccini and Perez, 2001; Ralet et al., 2001; Willats et al., 2001). When methyl groups of HG pectin are hydrolyzed by PME activity, Ca^2+^ can bind to the carboxylate ions, and crosslinking occurs. Extensive Ca^2+^ crosslinking occurs after completion of cell elongation, preventing further stretching. Pectins are major determinants of the porosity of cell walls and the middle-lamella, and the formation of an orderly pectic gel decreases the pore size of the pectin cell wall matrix and thus limits movement of water molecules (Baron-Epel et al., 1988; Wisniewski et al., 1991b; Rajashekar and Lafta, 1996). During the cold acclimation of grape cell cultures, pore size of the cell walls decreased from 35 to 22 Å (Rajashekar and Lafta, 1996). A pore size of 32 Å was found to restrict ice formation above -25°C (Gunnink and El-Jayyousi, 1993; Braccini and Perez, 2001). Limitation of water penetration into the cell walls will in turn limit the availability of water for transmembrane transport into the cells.

Long-distance transport of water toward the shoot tip occurs in the xylem, and lateral movement occurs through xylem pits. Transmembrane water movement may occur by diffusion through the lipid layer or, more importantly so, by bulk flow through aquaporins. Aquaporins are water-selective channel proteins in the cell membranes (Johansson et al., 2000; Maurel et al., 2008). Within the basal part of the bud and bud trace of peach trees, aquaporin transcript levels increased at time points corresponding with the end of endodormancy, suggesting regulation of the number of water transport channels through the growth-dormancy cycle (Yooyongwech et al., 2008, 2009).

In this study, we aimed to investigate whether the crown region at the base of the winter bud acts as a barrier by preventing water influx and to determine whether this is associated with SD-induced bud development. Microscopy and spectroscopy techniques as well as studies of aquaporin expression were applied to investigate effect of photoperiod on the structure–function relationships and biochemical changes *in situ*.

## Materials and Methods

### Plant Materials

Seeds of Norway spruce [*Picea abies* L. (Karst)] (59°N, provenance CØ1 from Halden, Norway, seed lot 98063, Skogfrøverket^1^) were sown and seedlings grown at 18°C in a 3:1 mixture of fertilized peat:perlite as previously described (Holefors et al., 2009). The relative air humidity was adjusted to 0.5 kPa water vapor pressure deficit. The photon flux density of the main light period of 12 h was 180 μmol m^-2^ s^-1^ at 400–750 nm from high-pressure sodium lamps (General Electric, United States) supplemented with 8 μmol m^-2^ s^-1^ from incandescent lamps (Osram, Germany). To obtain LD conditions, the light period was extended to 24 h with low-intensity light from incandescent lamps (8 μmol m^-2^ s^-1^, Osram). After 8 weeks, a subset of plants was transferred to SDs with a 12 h photoperiod with light conditions as in the main light period of LD and 12 h darkness. All other conditions were the same as for LD. After 8 weeks of SD, the plants were again placed under the LD conditions. In 30 plants per treatment, the plant length was measured from the soil base in the pot to the shoot tip/apical bud or to the tip of the longest needle, and elongation growth was calculated. To determine which of the apical needles were the longest, these needles were carefully bent upward.

### Plant Tissue Preparation and Structure Examination

For anatomical/histological studies plant materials from five plants per daylength treatment were embedded in LR White resin (London Resin Company, England). Harvested shoot tips, terminal buds, and breaking buds (2–3 mm long) were immediately fixed in 1% formaldehyde solution and 0.025% glutaraldehyde in sodium phosphate buffer (PBS, pH 7.0) and vacuum infiltrated at room temperature for 1 h and thereafter kept at 4°C overnight. The fixed samples were washed with PBS buffer and dehydrated in a graded ethanol series (30%, 50%, 70% 90%, and 100%). The samples were then infiltrated for 4 h in 1:1 LR White:ethanol (v/v), followed in sequence by 12 h in 2:1 LR White:ethanol (v/v) and pure LR White for 3 days. Thereafter the plant materials were embedded in the LR white resin by polymerization at 60°C for 12 h. Embedded plant materials were sectioned into 1 μm-thick sections using an Ultracut microtome (Leica EM UC6, Germany). The sections were stained with toluidine blue O (Sigma–Aldrich, United States) to visualize cells and tissues. The stained sections were examined using a light microscope (Leica DM6B, Germany).

Furthermore, terminal buds/shoot tips of SD and LD-exposed plants were longitudinally cut using a razor blade, immersed in fixation solution and examined using a stereoscope (Leica M205, Germany). The longitudinally cut tissues were mounted on a cryo-scanning electron microscope stub (cryo-SEM stub) and held in position using a cryoglue compound (Sakura, Japan). The stubs with sections were transferred to a pre-frozen SEM (Zeiss EVO 50 EP, Germany), and examined under vacuum with the cryo chamber of the SEM cooled to -180°C. To study more detailed cell structures, ultrathin sections (70 nm thick) of the LR White-embedded plant materials were obtained using an Ultracut microtome (Leica EM UC6). The sections were mounted on formvar-coated copper 100 mesh-grids [Electron Microscopy Sciences (EMS), United States]. The sections were then stained with uranyl acetate and lead citrate before examination of the sections using a transmission electron microscope (TEM) (FEI Morgagni 268, United States).

### Immunoanalyses of Cell Wall Components

**FIGURE 1:**
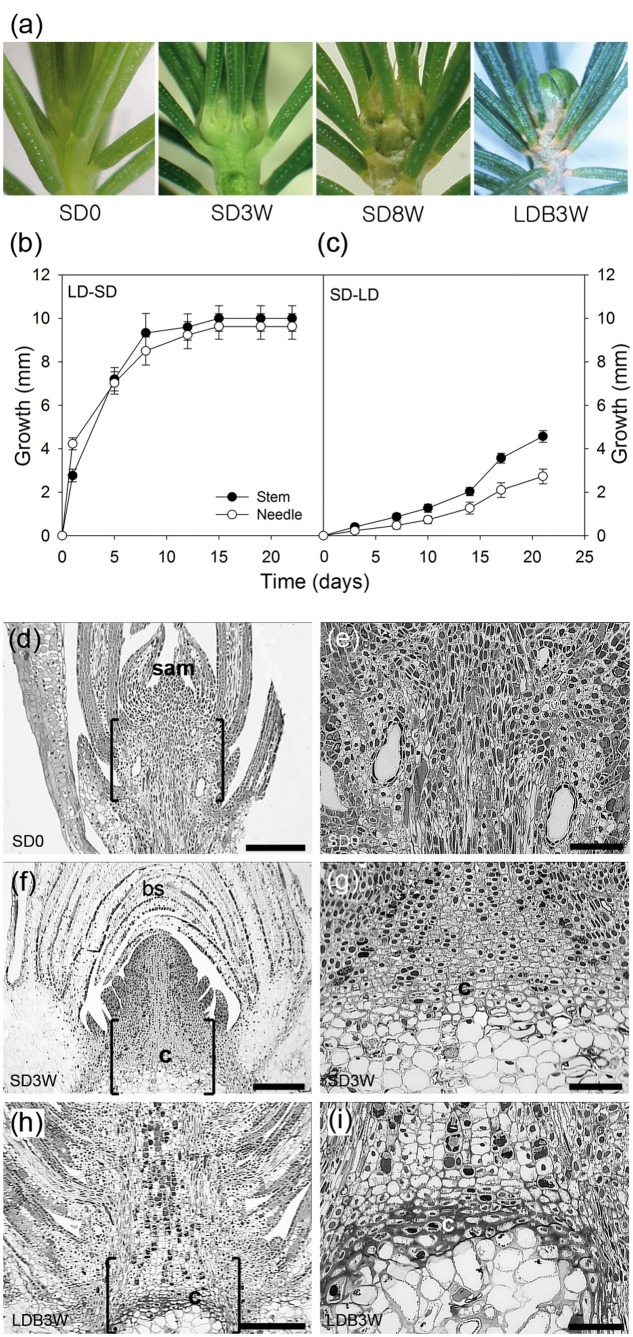
Effect of photoperiod on growth, morphology, and histology in shoot tips of Norway spruce seedlings. **(a)** Shoot tip morphology of (left to right) long day (LD)-grown seedlings (SD0 = no short days), after 3 (SD3W) and 8 weeks (SD8W) of SD-induced terminal bud development, and during terminal bud break 3 weeks after re-transfer to LD following SD8W (LDB3W). **(b)** Growth cessation after transfer from LD ( = time 0 = SD0) to SD and **(c)** re-initiation of growth after re-transfer to LD after SD8W. Growth was calculated on basis of distance between the soil base and either the shoot tip or tip of the longest needle. The values are mean ± SE of 30 plants per treatment. **(d–i)** Histology of shoot tip (**e**,**g**,**i** = higher magnification of area in brackets in **d**,**f**,**h**, respectively) with growing shoot tips at SD0 (**d** = overview, **e** = subapical region), terminal bud at SD3W (**f** = overview, **g** = crown region at the base of the bud), and breaking bud at LDB3W (**h** = overview, **i** = crown). sam, shoot apical meristem; bs, bud scales; c, crown. Scale bars: 310 μm **(d,f,h)**, 100 μm **(e,g,i)**.

The rat monoclonal antibodies JIM5 against de-methyl-esterified HG and JIM7 against methyl-esterified HG were used in this study (Clausen et al., 2003; Lee et al., 2008). For indirect immunofluorescence labeling, 1 μm thick sections of LR White-embedded material were obtained as described above. The sections were incubated in milk protein (MP)/PBS (pH 7.2) for 30 min to block non-specific binding. The sections were then incubated with primary antibodies diluted 1:10 in MP/PBS, for at least 1 h at 20°C or overnight at 4°C. Thereafter, the sections were washed with several changes of PBS prior to incubation for 1 h at 20°C with the secondary antibody (anti-rat-IgG; whole molecule) linked to fluorescein isothiocyanate (FITC, Sigma–Aldrich), diluted 1:100 in MP/PBS. The sections were then washed in PBS, mounted with Citifluor AF1 (EMS, United States) and examined using a microscope equipped with epifluorescence (Leica DM6B). The intensity of the fluorescence signal was assessed in similar areas in sections from five individual plants per daylength treatment and antibody (bound to its epitope) using ImageJ, and the values plotted as a densitogram. For this, the crown-region was used in SD and SD–LD-exposed plants as well as a region at the base of the shoot apical meristem in LD-grown plants as no such crown structure is present in these (area in brackets in **Figure 1**).

For immunoelectron microscopy (IEM), ultrathin sections (70 nm thick) were mounted on formvar and carbon-coated 100-mesh nickel grids (EMS, United States). The sections were blocked in 3% bovine serum albumin (BSA)/PBS. The sections were then treated for 1 h at 20°C with the primary JIM5 and JIM7 antibodies diluted 1:10 in BSA/PBS, and incubated for 1 h at 20°C in the secondary antibody solution containing anti-rat IgG coupled to 10 nm gold particles (Sigma–Aldrich), diluted 1:20 in BSA/PBS. The sections were then rinsed with PBS and stained with uranyl acetate and lead citrate before examining using a TEM (FEI Morgagni 268). The average number of gold particles were quantified in 30 different cell wall images from three different plants per daylength treatment.

To analyze callose deposition at plasmodesmata, mouse β-1,3-D-glucan antibody (Biosupplies, Australia) and anti-mouse IgG coupled to 10 nm gold particles (Sigma–Aldrich) were used as the primary and the secondary antibodies, respectively. After immunolabeling, the sections were stained with uranyl acetate and lead citrate before examination using the TEM. The average number of gold particles were quantified in 100 different plasmodesmata from three different plants per daylength treatment.

### Ca^2+^ Localization

To study Ca^2+^ localization in cell walls in the crown region, plant materials from five plants per daylength treatment were vacuum infiltrated for 1 h in fixative solution consisting of 5% (w/v) potassium pyroantimonate [(K_2_H_2_Sb_2_)7⋅4H_2_O] and 2% (w/v) osmium tetroxide at pH 7.5, and placed at 4°C for 12 h. As described above, fixed plant tissues were dehydrated and embedded in LR White resin, and ultrathin sections were made and mounted on 100-mesh formvar coated copper grids and stained with uranyl acetate and lead citrate. The pyroantimonate precipitate present in the ultrathin sections was examined using the TEM (FEI Morgagni 268).

### Mid-Infrared Analysis of Cell Wall Components

For analysis of the presence and distribution of cell wall components *in situ* in the crown region of SD-exposed plants and plants exposed to SD followed by LD, frozen bud/shoot tip samples were longitudinally cut into 6 μm thick sections using a cryo-microtome (MICROM, Germany). The sections were deposited on 3 mm thick barium fluoride (BaF_2_) slides (Crystran, United Kingdom).

Unstained sections were analyzed using mid-infrared (mid-IR) spectroscopy combined with microscopy (spectromicroscopy) (Aspinall, 1970; Budevska, 2006) at the Canadian Light Source Inc, synchrotron (beamline 01B1-1; Saskatoon, SK, Canada).

Identification of HG pectin was done on basis of characteristic peaks of polygalacturonic acid (1,736, 1,330, 1,397, 1,220, 1,147, 1,017, 952, 890, and 830 cm^-1^) that are distinct from other common cell wall components like lignin, cellulose, and hemicellulose (Chatjigakis et al., 1998; Copikova et al., 2001; Karunakaran et al., 2015). Two distinct IR vibrational bands were used to differentiate highly methyl-esterified and de-methyl-esterified pectin: 1,749 cm^-1^ (C=O vibration of ester) and 1,630 cm^-1^ (COO^-^ antisymmetric vibration), respectively (Chatjigakis et al., 1998; Copikova et al., 2001; Szymanska-Chargot and Zdunek, 2013). Other bands with strong vibrations were also used to determine the methylation status of the HG pectin: 1,440 cm^-1^ (CH_3_ group in the ester), 1,410 cm^-1^ (COO^-^ symmetric vibration of ester), 954 cm^-1^ (C–O bending), and 832 cm^-1^ (ring vibration) based on pure pectin spectra (Szymanska-Chargot and Zdunek, 2013). To determine the relative quantities of the HG pectin forms in the samples, IR map data were generated by integrating the regions between 1,830 and 1,695 cm^-1^ (methyl-esterified carboxyl group; with the main peak at 1,749 cm^-1^) and 1,695–1,570 cm^-1^ (non-esterified carboxyl group; with the main peak at 1,630 cm^-1^) (Chatjigakis et al., 1998). Also, vibrational bands representing proteins were inspected (Szymanska-Chargot and Zdunek, 2013). The 1,690–1,600 cm^-1^ band (with the main peak at 1,655 cm^-1^) represents the C=O stretching from the amide I vibration, and the 1,575–1,480 cm^-1^ band (with the main peak at 1,545 cm^-1^) represents the CN stretching and NH bending from the amide II vibration.

For higher resolution analysis of selected spots on the samples, a Bruker Vertex 70v/S spectrometer with a Hyperion 3000 microscope (Bruker Optics, Ettlingen, Germany) was used first with a mercury cadmium telluride (MCT) detector and the synchrotron mid-IR beam (spatial resolution range 3–15 μm, signal-to-noise ratio 100–1,000). The mid-IR data were collected in the transmission mode at a spectral resolution of 4 cm^-1^ in the 4,000–800 cm^-1^ wavelength range. A light microscope equipped with a camera was used to identify regions of interest. The sample chamber was purged with dry N_2_ to minimize IR absorption by water vapor and CO_2_. Spectra from selected regions of the sections were first collected at a spatial resolution of 15 μm × 15 μm using the MCT detector. Totally 64 co-added scans were recorded at each spot on the sample and the average of the scans was corrected using a background spectrum (average of 128 scans) obtained from an area where there was no sample.

The same samples were then used to collect large area maps using a globar source (larger, uniform beam) and a 64 × 64 pixel focal plane array (FPA) detector (spatial resolution about 2.7 μm × 2.7 μm). The spectra of samples were first baseline corrected using the rubber-band correction (64 points, version 7.2, Bruker Optics Inc., Billerica, MA, United States). The second derivative spectral processing technique (using graphing software OriginPro, version 2015) was then used to enhance the separation of overlapping peaks in the original spectra. This allowed more specific identification of small absorption peaks (Rieppo et al., 2012) of the cell wall components described above (methyl-ester group of methyl-esterified HG and carboxyl group of de-methyl-esterified HG as well as amide I and amide II bands of proteins). Also, on basis of these second derivative spectra, the degree of methyl-esterification of the HG pectin in the samples (% methyl-esterified HG of total HG) was determined according to the following (Chatjigakis et al., 1998): Number of methyl-esterified carboxylic groups/total number of carboxylic groups) × 100]. This is proportional to: Area of the peak at 1,749 cm^-1^/(area of the peak at 1,749 cm^-1^ + area of the peak at 1,630 cm^-1^). The limits of the peak at 1,749 cm^-1^ were set from 1,830–1,695 cm^-1^ and those of the peak at 1,630 cm^-1^ from 1,695–1,570 cm^-1^.

### Phloroglucinol, Fluorescein, and Laurdan Staining

Since the cell walls of xylem tracheids are lignified (Sarkar et al., 2009), xylem distribution across the crown region was visualized in longitudinal sections of fresh shoot tips, terminal buds and breaking buds stained for lignin with 10% (w/v) phloroglucinol in 95% ethanol for 10 min. An equal volume of concentrated HCl was added and the sections left for 2–3 min. The sections were then rinsed thoroughly with distilled water and examined using a light microscope (Leica DM6B).

To visualize water uptake, shoot tips of Norway spruce seedlings were cut in approximately 2 cm length from the shoot tip or bud tip and immersed into a 0.1% (w/w) solution of sodium fluorescein (Sigma–Aldrich) for 30 min. After immersion, the cut side of the plants was briefly rinsed with distilled water, followed by longitudinal dissection, and examination under a light microscope equipped with epifluorescence detection (Leica DM6B).

To further visualize (indirectly) the penetration of water into the tissues, laurdan (6-dodecanoyl-2-dimethylaminonaphthalene, Sigma) dissolved in 60 mM DMSO containing 17% ethanol was used in the same experimental procedure as for the fluorescein immersion. Laurdan has a high sensitivity to mobility and presence of solvent dipoles such as water molecules, and is sensitive to membrane phase transitions and alterations in membrane fluidity due to penetration of water (Sanchez et al., 2012). For all of these staining experiments, 10–15 individual plants were used per daylength treatment.

### Determination of Water and Dry Matter Content

The content of water and dry matter were measured in a time course in 5 mm long parts of shoot tips of growing plants under LD, in developing buds after transfer to SD and in breaking buds after re-transfer to LD following 8 weeks of SD. Bud scales were removed before determination of fresh weight and lyophilization. Dry matter was then determined using an electronic microbalance, and water content calculated.

### Aquaporin mRNA Localization *in Situ* and Analysis of Transcript Levels

For localization of aquaporin mRNA *in situ* and analysis of aquaporin transcript levels, shoot tips from growing plants under LD, terminal buds after 8 weeks of SD, and breaking buds 1–4 weeks after re-transfer to LD following 8 weeks under SD, were harvested.

For the *in situ* localization, the plant materials were immediately fixed in formaldehyde solution, washed and dehydrated as described above. The tissues were then embedded in paraplast (Sakura, Japan) using a Tissue-Tek VIP Jr. automatic embedding machine (Sakura, Japan). Single-stranded RNA probes were synthesized after linearization of the pCR4-TOPO plasmid DNA (Invitrogen, United States) carrying an aquaporin gene (*PaAQP*; GenBank accession no AY961921.1), and digestion using NotI and SpeI restriction enzymes for sense and antisense probes, respectively. Sense and antisense probes, labeled with digoxigenin-dUTP using a DIG RNA labeling kit (Roche, Germany), were prepared using T3 and T7 RNA polymerases. The 10 μm-thick paraplast-embedded sections were cut using a rotary microtome (Leica LM2255, Germany), and placed on poly-L-lysine coated glass slides and left at 42°C overnight. The sections were de-waxed using Histoclear (Cell path, England) and rehydrated in a graded ethanol-series. The tissues were then placed in a proteinase K (1 μg ml^-1^) solution at 37°C for 30 min, acetylated using 0.5% acetic anhydride in 0.1 M triethanolamine, followed by washing in PBS solution and dehydration. The 100 ng labeled anti-sense and sense riboprobes in 40 μl hybridization solution were applied to each slide and incubated in a humid chamber at 50°C for 16 h. The hybridization solution contained 50% formamide, dextran sulfate, Denhardt’s solution, tRNA and hybridization buffer. After hybridization, the sections were washed with 3x SSPE buffer at 50°C. The sections were then treated with RNase in NTE buffer (0.5M NaCl, 10 mM Tris-HCl pH 7.5, and 1 mM EDTA) at 37°C for 30 min to remove unbound RNA probes, and slides were washed twice in preheated NTE buffer with 15 min intervals, followed by two times washing with 2x SSPE and 0.1x SSPE buffer at 60°C. The sections were then blocked using 1% Boehringer blocking reagent (Roche, Germany). The hybridized probes were detected immunologically using anti-digoxigenic-alkaline phosphate-coupled antibody diluted 1:3,000 in 1% blocking reagent. Sections were visualized by applying 5-bromo-4-chloro-3-indolyl phosphate (BCIP) and nitroblue tetrazolium salt (NBT) diluted in alkaline phosphate buffer, and the color reaction was developed in the dark at room temperature. The slides were finally washed in water and stop solution followed by dehydration in ethanol series, and then mounted in DePeX (Sigma–Aldrich, United States). Images were taken using a light microscope with bright field optics (Leica DM6, Germany).

For analyses of aquaporin transcript levels shoot tip/bud materials were harvested and dissected into three parts, i.e., bud (ca 2–3 mm), crown (ca 1 mm junction between bud and stem), and 5 mm stem tissue below the bud and crown. Since there were no bud and crown structure in the LD-grown plants, the upper needles were removed and the shoot tips divided into the following samples: (1) the upper 2–3 mm containing the apical meristem with leaf initials, (2) 1 mm stem tissue beneath this, and (3) 5 mm stem tissue beneath this 1 mm. In each case, three repeated samples were harvested, each consisting of materials from 8 to 10 plants (shoot tip/buds and stem) and 15 to 20 plants (crown). The samples were harvested into liquid nitrogen and stored at -80°C until analyses. Total RNAs were isolated using MasterPure^TM^ Plant RNA Purification Kit (Epicentre, United States) following the manufacturer’s instructions. Residual DNA was removed using RNase free DNase treatment and RNA was purified using ethanol precipitation and PureLink RNA Mini-kit (Ambion, United States) as described by the manufacturer. Quality and quantity of isolated RNA were analyzed using an Agilent 2100 Bioanalyzer with an RNA 6000 NanoKit (Agilent Technologies, Germany). cDNA was synthesized from 1 μg of total RNA using the Taqman Reverse Transcription Reagents (Applied Biosystems, United States).

The RT-qPCR (quantitative real-time PCR) analyses were conducted using the SuperScript III Platinum Two-Step qRT-PCR Kit with SYBR GreenER master mix (Invitrogen, United States). The amplification was performed with a 7500 Real-time PCR system (Applied Biosystems, United States) in a 25 μl reaction volume using 2 μl diluted cDNA solution as template, 12.5 μl SYBR GreenER master mix (Invitrogen, United States) and 200 nm of each primer. RT-qPCR Reactions were done in triplicate for each sample and a no-template control was run for each primer pair. The following program was used for amplification: 95°C for 2 min followed by 45 cycles of 95°C for 15 s and 60°C for 35 s. After amplification, a melting curve analysis was carried out. Target gene expression was normalized to Norway spruce *α-TUBULIN* (Sundas et al., 1992). The primers used for the RT-qPCR analysis were the following; for the *AQUAPORIN* gene *PaAQP* (GenBank accession no. AY961921.1) forward: CGCGGGGTAGCCATCATTTA, reverse: ATGGCTCGGATGATGAGCTG, and for the *α-TUBULIN* gene (GenBank accession no. X57980) forward: CTGGAACCCACGGTCATT, reverse: ACCACGAGCGAAGTTGTTG.

### Statistical Analyses

The quantitative results were analyzed by one-way or two-way analysis of variance (ANOVA, General linear model), followed by Tukey’s multiple comparison test (*p* ≤ 0.05) (Minitab Version 16, Minitab Inc., United States). Two-way ANOVA was used to analyze the abundance of JIM5 and JIM7 epitopes in cell walls in the crown region of plants exposed to different daylength treatments, For the fluorescence recordings, five plants per daylength treatment and epitope were used and for the gold particle recordings, 30 different cell wall images from each of three plants per daylength and epitope. One-way ANOVA was used to analyze the number of gold particles in the study of callose deposition at plasmodesmata in the crown region with 100 different plasmodesmata from three plants per daylength treatment. Water content and dry matter in shoot tips/buds were analyzed by one-way ANOVA using three plants per daylength treatment in each case. Effect of daylength treatment on transcript levels of an aquaporin gene in different plant parts (crown, bud and stem) were analyzed by two-way ANOVA using three repeated samples, each consisting of 8–10 plants (bud and stem) or 15–20 plants (crown) per daylength treatment.

## Results

### SD-Induced Terminal Buds Have a Crown Region of Thick-Walled Cells at the Base

As expected, shoot elongation was sustained under LD and after transfer to SD, growth cessation occurred after about 14 days and a distinct green, terminal bud was visible after 3 weeks (**Figures 1a,b**). After 8 weeks of SD treatment, well-developed terminal buds with brown, layered bud scales were present. After re-transfer to LD following 8 weeks under SD, bud break and re-initiation of shoot growth was apparent within 3 weeks (**Figures 1a,c**).

**FIGURE 2:**
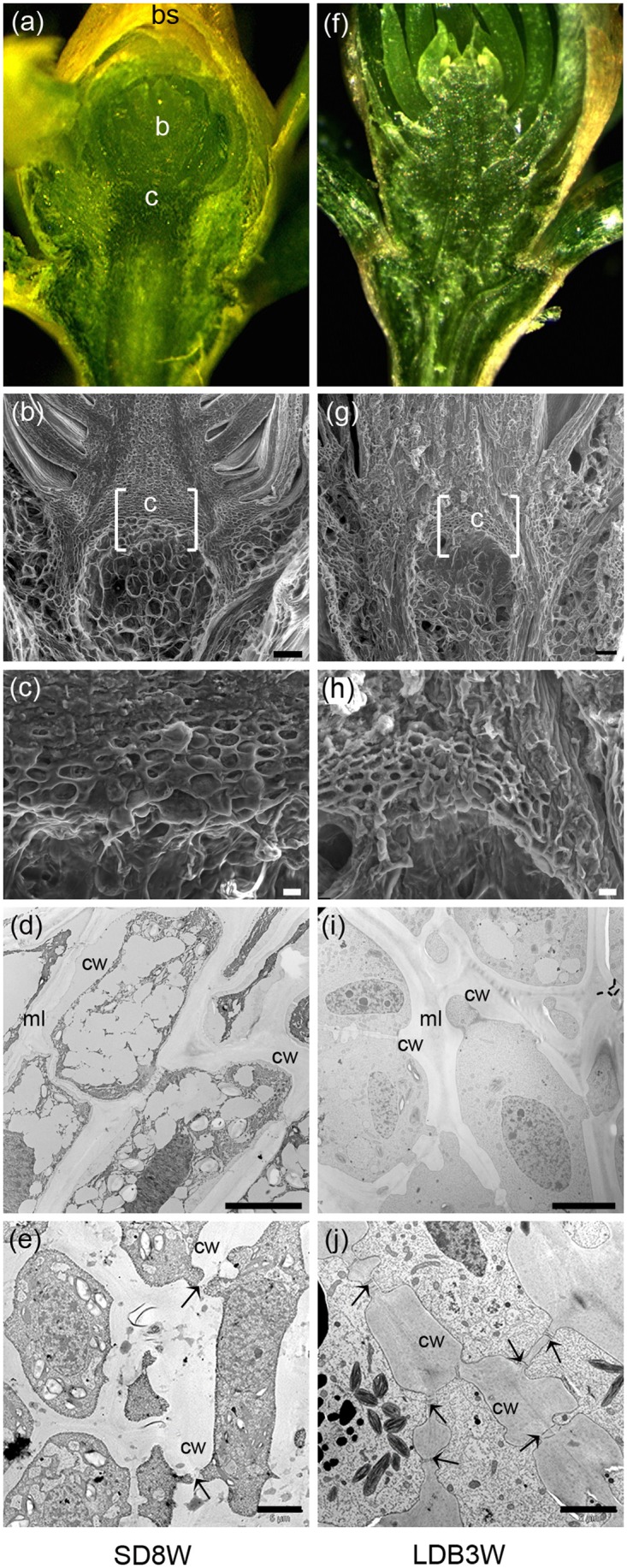
Morphology, histology, and subcellular structures of a terminal bud after 8 weeks of short day (SD) treatment [SD8W **(a–e)**] and a breaking bud 3 weeks after re-transfer to LDs following SD8W [LDB3W **(f–j)**] in Norway spruce seedlings. **(a)** Longitudinal section of a terminal bud. **(b)** Scanning electron microscope (SEM) image of a sectioned terminal bud. **(c)** Higher magnification of the crown region shown in brackets in **b**. **(d,e)** Transmission electron microscope (TEM) images (different magnifications) of cells in the crown region. **(f)** Longitudinal section of a breaking bud. **(g)** SEM image of a sectioned breaking bud. **(h)** Higher magnification of the crown region in **g**. **(i,j)** TEM images of cells in the crown region of a breaking bud. b, bud; bs, bud scale; c, crown; cw, cell wall; ml, mid-lamella. Arrows indicate plasmodesmata. Scale bars: 100 μm **(b,g)**, 20 μm **(c,h)**, 10 μm **(d,i)**, 5 μm **(e,j)**.

In contrast to elongating plants under LD (**Figures 1d,e**), in plants exposed to SD for 3 weeks, the cell plate of radially elongated thick-walled cells, denoted the crown region, was visible across the pith at the base of the terminal bud (**Figures 1f,g**). When re-initiation of growth was visible 3 weeks after re-transfer to bud-break-inducing LD following 8 weeks of SD, the crown cells were very distinct with thick cell walls (**Figures 1h,i**). Further studies using SEM and TEM, confirmed the presence of a distinct crown region with thick cell walls and revealed a broad middle-lamella after 8 weeks under SD (**Figures 2a–e**) as well as 3 weeks after the re-transfer to LD (**Figures 2f–j**).

### SD-Induced Modification of Cell Wall Pectin in the Crown Region

**FIGURE 3:**
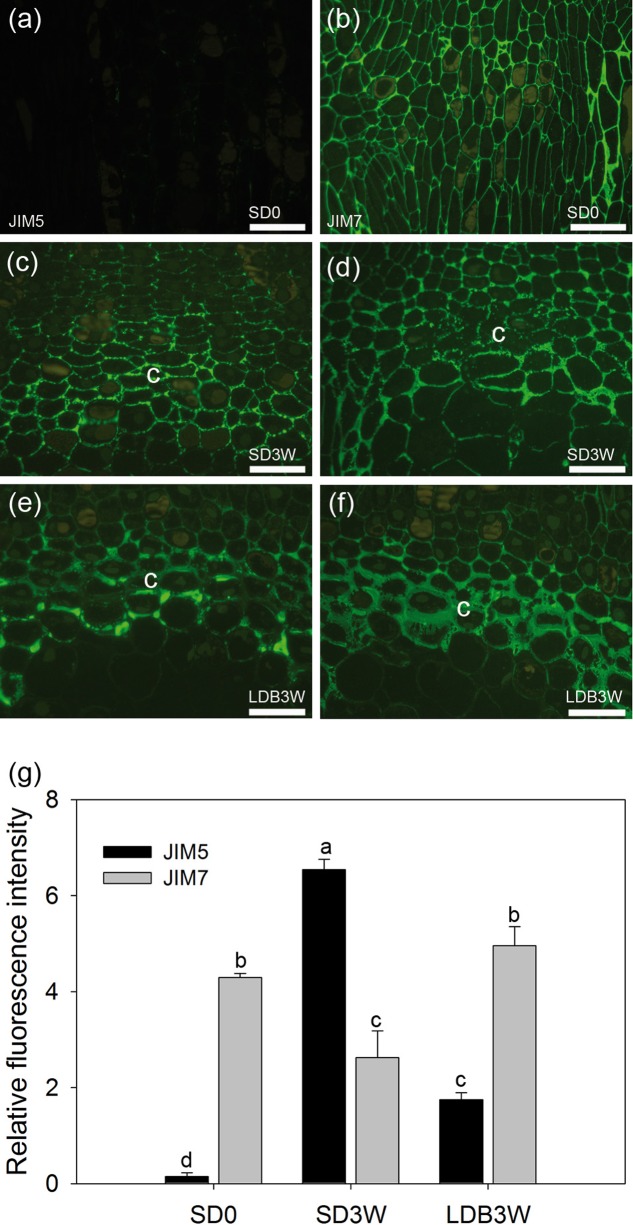
Effect of photoperiod on methyl-esterification status of homogalacturonan (HG) pectin in shoot tips of Norway spruce seedlings as analyzed by immunolocalization. Micrographs of indirect immunolocalization of **(a,c,e)** de-methyl-esterified JIM5 and **(b,d,f)** methyl-esterified JIM7 HG epitopes in longitudinal sections of growing shoot tips under long days (LD = time 0 before transfer to short days = SD0; **a**: JIM5, **b**: JIM7), in the crown region at the base of a terminal bud after 3 weeks of SD treatment (SD3W; **c**: JIM5, **d**: JIM7), and in a breaking bud 3 weeks after re-transfer to LD after 8 weeks of SD treatment (LDB3W; **e**: JIM5, **f**: JIM7). c, crown. Scale bars: 50 μm (Light micrographs of the histological sections are shown in **Figures 1e,g,i**). **(g)** Densitogram of the fluorescence signal intensity of JIM5 and JIM7. The values are mean ± SE of sections from five plants per daylength treatment and epitope. Different letters indicate significant differences.

To investigate the methyl-esterification status of the main cell wall pectin HG, indirect immunolabeling followed by densitometric scanning of the fluorescence signal intensity was employed (**Figure 3**). In shoot tips of growing plants under LD, there was virtually no detectable binding of the JIM5 antibodies against de-methyl-esterified HG (**Figures 3a,g**). In contrast, the JIM7 antibodies against methyl-esterified HG showed extensive binding under LD (**Figures 3b,g**). After 3 weeks of SD exposure, there was extensive binding (significant increase compared to LD, *p* ≤ 0.05) of the JIM5 antibodies in the cell walls of the crown region, but not in other areas of the shoot apical meristem (**Figures 3c,g**). There was still abundant JIM7 antibody binding after 3 weeks under SD but the occurrence of the JIM7 HG epitope was then slightly but significantly reduced in the crown area (**Figures 3d,g**). In comparison, in plants showing bud-break after 3 weeks under LD following 8 weeks of SD there was significantly lower and higher intensity of JIM5 HG (**Figures 3e,g**) and JIM7 HG labeling (**Figures 3f,g**), respectively.

**FIGURE 4:**
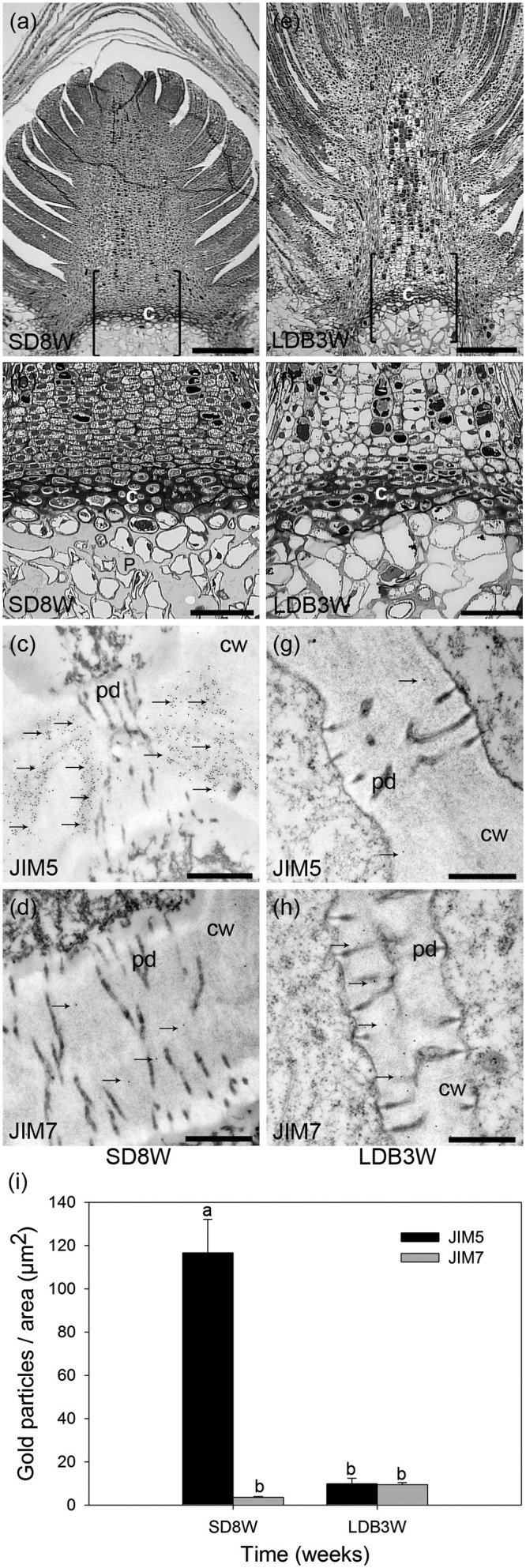
Effect of photoperiod on methyl-esterification status of HG pectin in the cell walls of the crown region at the base of the terminal bud of Norway spruce seedlings as analyzed by immunoelectron detection in transmission electron microscopy (TEM). **(a–d)** Terminal buds after 8 weeks under SDs (SD8W). **(e–h)** Breaking buds 3 weeks after re-transfer to LDs following SD8W (LDB3W) (Light micrographs in **a** and **e** are shown in higher magnification in **b** and **f**, respectively). **(c,d,g,h)** Transmission electron micrographs of gold nanoparticle immunoelectron detection of de-methyl-esterified JIM5 (**c**: SD8W, **g**: LDB3W) and methyl-esterified JIM7 (**d**: SD8W, **h**: LDB3W) HG epitopes in the crown region at SD8W and LDB3W. c, crown; cw, cell wall; p, pith; pd, plasmodesmata. Arrows indicate gold particles bound to the antibodies. Scale bars: 310 μm **(a,e)**, 100 μm **(b,f)**, 500 nm **(c,d,g,h)**. **(i)** The average number of gold particles in the sections. The values are mean ± SE of 30 different cell wall images in sections from three different plants per daylength treatment and epitope. Different letters indicate significant differences.

For more detailed localization and quantification of the HG epitopes in cell walls gold nanoparticle IEM was performed (**Figure 4**). After 8 weeks of SD, when the plants had a well-developed crown structure and disorganized cells in the pith area beneath the crown (**Figures 4a,b**), there were abundant JIM5 HG epitopes (de-methyl-esterified HG) in the cell walls of the crown region (**Figures 4c,i**). In contrast, the abundance of JIM7 HG epitopes (methyl-esterified HG) was then significantly lower in this region (**Figures 4d,i**). Three weeks after re-transfer to LD following 8 weeks of SD (**Figures 4e–h**), the cell walls in the crown structure of the breaking buds appeared to have similar or possibly a slight trend of increased levels of JIM7 HG epitopes as compared to after 8 weeks of SD (**Figures 4h,i**). However, significantly lower abundance of JIM5 HG epitopes (*p* ≤ 0.05) was detected as compared to the response after 8 weeks of SD (**Figures 4g,i**).

**FIGURE 5:**
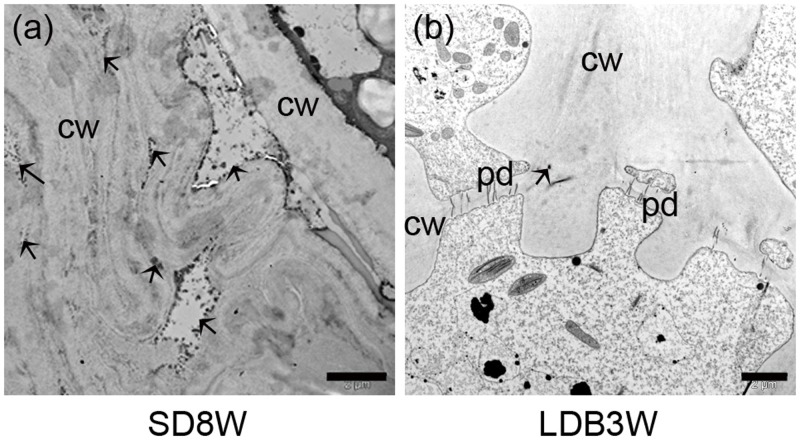
Histochemical localization of Ca^2+^ in cell walls and middle lamella of the grown region at the base of terminal buds in Norway spruce seedlings, visualized as potassium pyroantimonate- Ca^2+^ complex (dark spots/areas with black spots indicated by arrows). **(a)** Terminal bud after SDs for 8 weeks (SD8W). **(b)** Breaking bud 3 weeks after re-transfer to LDs after SD8W (LDB3W). cw, cell wall; pd, plasmodesmata. Scale bars: 2 μm.

Since de-methyl-esterified HG, in contrast to methyl-esterified HG, can bind Ca^2+^, histochemical localization of Ca^2+^ was also performed. After 8 weeks of SD, the cell walls and mid-lamella of the crown region showed high abundance of Ca^2+^ (**Figure 5a**). In contrast, very little Ca^2+^ was observed in breaking buds 3 weeks after re-transfer to LD following 8 weeks of SD (**Figure 5b**).

### Mid-IR Analysis Supports Photoperiodic Control of Cell Wall Composition

**FIGURE 6:**
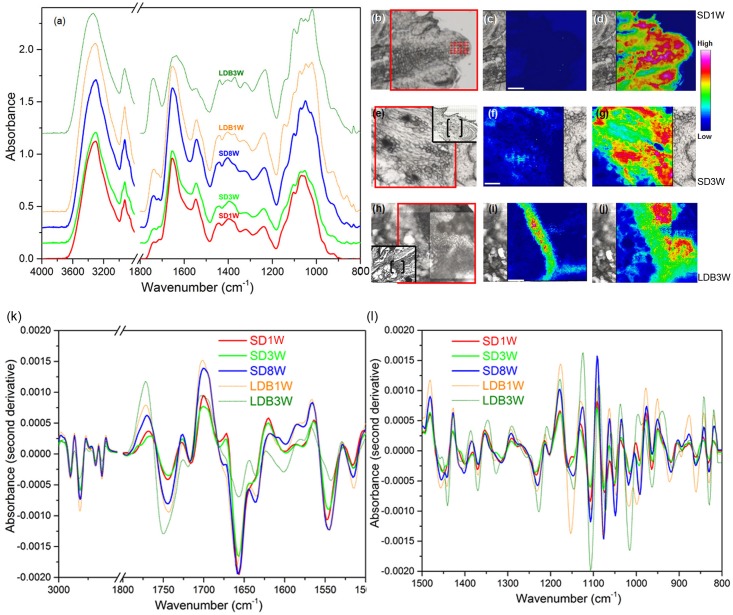
Mid-infrared analysis of cell wall components. **(a)** Absorbance spectra for cell wall components in cells of the crown region of plants exposed to bud set-inducing SDs for 1 (SD1W; no distinct crown; shoot apex analyzed), 3 (SD3W) or 8 (SD8W) weeks, and during bud break 1 (LDB1W) or 3 (LDB3W) weeks after re-transfer to LDs following SD8W. **(b,e,h)** Micrographs of the SD1W, SD3W, and LDB3W samples used to evaluate the relative intensity of methyl-esterified and de-methyl-esterified HG pectin (area within red frame). In **(e,h)**, a larger overview of the section is shown within the black frame (bud scales/shoot tip to the right in all micrographs/photos) with the analyzed area in brackets. **(c,f,i)** Intensity of the methyl-ester group (1,830–1,695 cm^-1^ with the main peak at 1,749 cm^-1^) vibration (methyl-esterified HG pectin), and **(d,g,j)** the carboxyl group (1,695–1,570 cm^-1^ with the main peak at 1,630 cm^-1^) vibration (de-methyl-esterified HG pectin) in the SD1W, SD3W, and LDB3W samples. **(k,l)** Second derivative absorbance spectra for enhanced peak separation of cell wall components in the same samples, i.e., methyl-esterified and de-methyl-esterified HG (bands as mentioned) and proteins (amide I and amide II vibration bands at 1,690–1,600 cm^-1^ (the main peak at 1,655 cm^-1^) and 1,575–1,480 cm^-1^ (the main peak at 1,545 cm^-1^), respectively).

To further characterize the effect of photoperiod on cell wall composition, mid-IR spectroscopy was performed (**Figure 6**). The samples of the crown region of plants exposed to SD for 8 weeks, had the strongest vibration of a main peak for de-methyl-esterified HG pectin; 1,630 cm^-1^, followed by those exposed to 1 and 3 weeks of SD (**Figure 6a**). After the re-transfer to LD following 8 weeks of SD, there was a shift toward higher wavenumbers characteristic of higher degree of methyl-esterification, i.e., with a main peak at 1,749 cm^-1^ (**Figure 6a**). Furthermore, as compared to the other samples, after 3 weeks of LD following the re-transfer from SD, stronger absorbance peaks were also observed for lower wavenumber bands characteristic for methyl-esterified HG (1,440, 1,370, 1,227, 1,014, 831 cm^-1^) (**Figure 6a**).

Integration of the areas of specific vibrational band regions characteristic of the methyl-esterified (1,830–1,695 cm^-1^ with the main peak at 1,749 cm^-1^) and de-methyl-esterified (1,695–1,570 cm^-1^ with the main peak at 1,630 cm^-1^) carboxylic group in HG pectin, revealed low content of methyl-esterified HG after 1 week under SD (**Figures 6b,c**), whereas the quantity of de-methyl-esterified HG was high (**Figures 6b,d**). The situation after 3 weeks of SD was very similar with low and high levels in the crown region of methyl-esterified and de-methyl-esterified HG, respectively (**Figures 6e–g**). Of the samples analyzed, plants grown 3 weeks after re-transfer to LD following 8 weeks under SD, had the highest abundance of methyl-esterified HG in the crown region (**Figures 6h,i**). In these, the content of de-methyl-esterified HG was reduced (**Figures 6h,j**) as compared to under SD (**Figures 6d,g**). On basis of the second derivative spectra, it was estimated that 7% and 35% of the cell wall HG was present as the methyl-esterified form in plants exposed to 1 and 3 weeks of LD, respectively, following 8 weeks of SD. In SD-exposed plants 2–5% of the HG was estimated to be in this form.

The spectra (**Figures 6a,k,l**) also revealed that the samples had strong absorbance bands in the amide I (1,690–1,600 cm^-1^ with the main peak at 1,655 cm^-1^) and amide II (1,575–1,480 cm^-1^ with the main peak at 1,545 cm^-1^) regions of proteins. The samples of plants exposed to SD for 8 weeks had considerably higher absorbance bands of amide I and amide II group groups than the samples harvested 3 weeks after the re-transfer to LD following 8 weeks of SD. The latter had the lowest absorbance bands of amide I and amide II groups compared to all the other samples.

### SD-Induced Callose Deposition at Plasmodesmata in the Cells of the Crown Region

**FIGURE 7:**
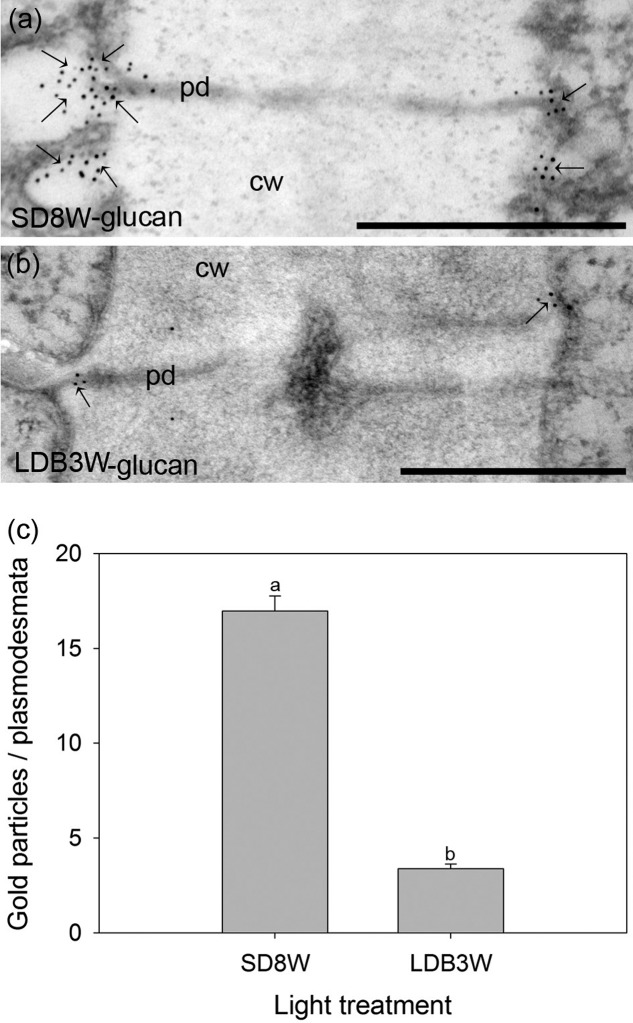
Gold nanoparticle immunoelectron localization of callose (β-1,3-D-glucan) at plasmodesmata in the crown region at the base of the terminal bud **(a)** after 8 weeks of SD exposure (SD8W) and **(b)** in breaking buds 3 weeks after re-transfer to LDs following SD8W (LDB3W). cw, cell wall; pd, plasmodesmata. Arrows indicate gold particles. **(c)** The average number of gold particles on the plasmodesmata in the SD8W and LDB3W sections. The values are mean ± SE of 100 different plasmodesmata from three plants per treatment. Different letters indicate significant difference. Scale bars: 500 nm.

To evaluate whether the cells in the crown structure at the base of the terminal bud are connected through plasmodesmata, localization of β-1,3-D-glucan (callose) was performed using gold-nanoparticle labeled antibodies. In plants exposed to SD for 8 weeks, significantly more gold particles were observed at the plasmodesmata than in breaking buds 3 weeks after re-transfer to LD following 8 weeks of SD (**Figure 7**).

### Discontinued Xylem Connections across the Crown Region under SD

**FIGURE 8:**
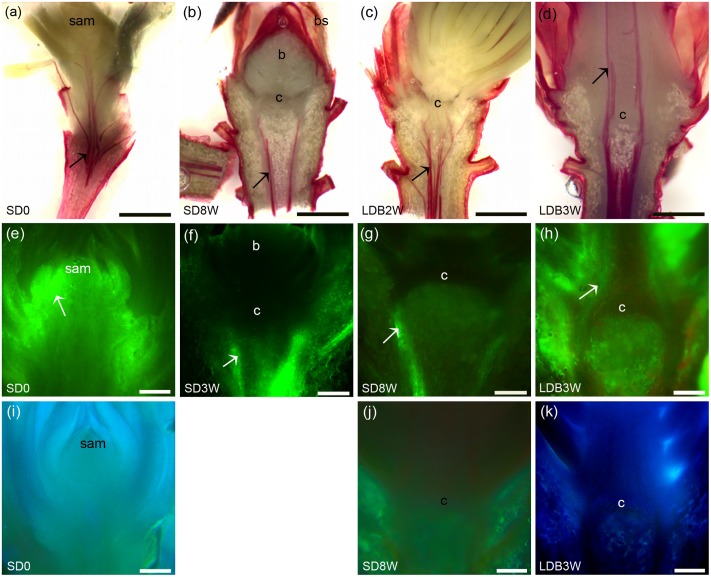
Effect of photoperiod on lignin and water uptake/localization in shoot tips/terminal buds of Norway spruce seedlings. **(a–d)** Phloroglucinol staining of lignin (red color): **(a)** Shoot tip of LD-grown seedling (= 0 short days = SD0), **(b)** terminal bud after 8 weeks under SDs (SD8W), **(c,d)** breaking bud 2 (**c**; LDB2W) and 3 (**d**; LDB3W) weeks after re-transfer to LD following SD8W treatment. **(e–h)** Fluorescein staining (uptake experiment): **(e)** Shoot tip of growing seedling (SD0), **(f,g)** terminal buds at SD3W and SD8W, **(h)** breaking bud at LDB3W. White arrows indicate strong fluorescein signal and discontinuation of the signal at the crown structure under SD **(f,g)**. **(i–k)** Laurdan staining for evaluation of penetration of water into the tissues: **(i)** Shoot tip of growing seedling (SD0), **(j)** terminal bud at SD8W, **(k)** breaking bud at LDB3W. c, crown region (at the base of the bud). Scale bars: 1 mm **(a–d)**, 200 μm **(e–k)**.

The presence of xylem connections across the crown region into the terminal bud was evaluated by staining of lignin. Under LD, plants stained for lignin in the cell walls of the xylem up to the shoot tips (**Figure 8a**). However, after 8 weeks of SD, such lignin staining was observed only beneath the crown structure at the base of the bud and not inside the terminal bud or within the crown (**Figure 8b**). Two weeks after re-transfer to LD after 8 weeks under SD, lignin staining into the crown was again observed (**Figure 8c**), and after 3 weeks continuous lignin staining up to the shoot tip was observed in the breaking buds (**Figure 8d**).

To assess the possibility for water transport into shoot tips of growing plants under LD, in SD-induced terminal buds, and upon LD-induced bud break, fluorescein uptake experiments were performed. In the LD grown plants, the fluorescein solution was taken up into the shoot tips and the fluorescence was visible throughout the shoot primordia (**Figure 8e**). However, after 3 weeks of SD, the fluorescence was only observed beneath the crown structure, and not in the crown region or within the developing terminal bud (**Figure 8f**). After 8 weeks of SD, the terminal buds and the crown structure did also not show any fluorescence, and fluorescence was limited to the region beneath the crown (**Figure 8g**). In contrast, 3 weeks after re-transfer to LD, fluorescein was observed in the breaking buds where re-initiation of growth occurred (**Figure 8h**). In supplementary experiments evaluating the possibility for penetration of water into the tissue by laurdan staining, similar results were observed. Staining was observed throughout the shoot apex under LD in contrast to under SD when staining was limited to the region beneath the crown (**Figures 8i–k**).

**FIGURE 9:**
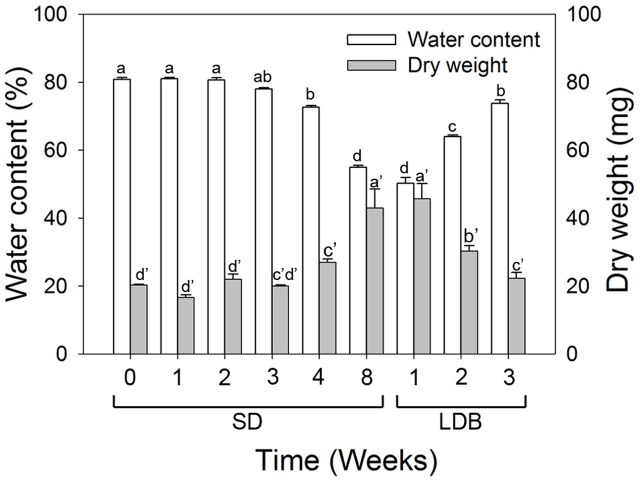
Water content and dry matter of shoot tips of long day (LD = 0 short days = SD0) grown Norway spruce seedlings, during SD-induced terminal bud development, and subsequent LD-induced bud-break (LDB) after re-transfer to LD following 8 weeks of SD treatment. The values are mean ± SE of thee plants per daylength treatment and time point. Different letters within one parameter indicate significant differences.

The water content and dry matter was gradually reduced and increased, respectively, (*p* ≤ 0.05) in the shoot tips during SD-induced terminal bud development as compared to LD (**Figure 9**). The situation was reversed with increasing water content and decreasing dry matter (*p* ≤ 0.05) after re-transfer from SD to LD.

### Aquaporin Transcript Levels Increase in Stem Tissue after Re-transfer to LD

**FIGURE 10:**
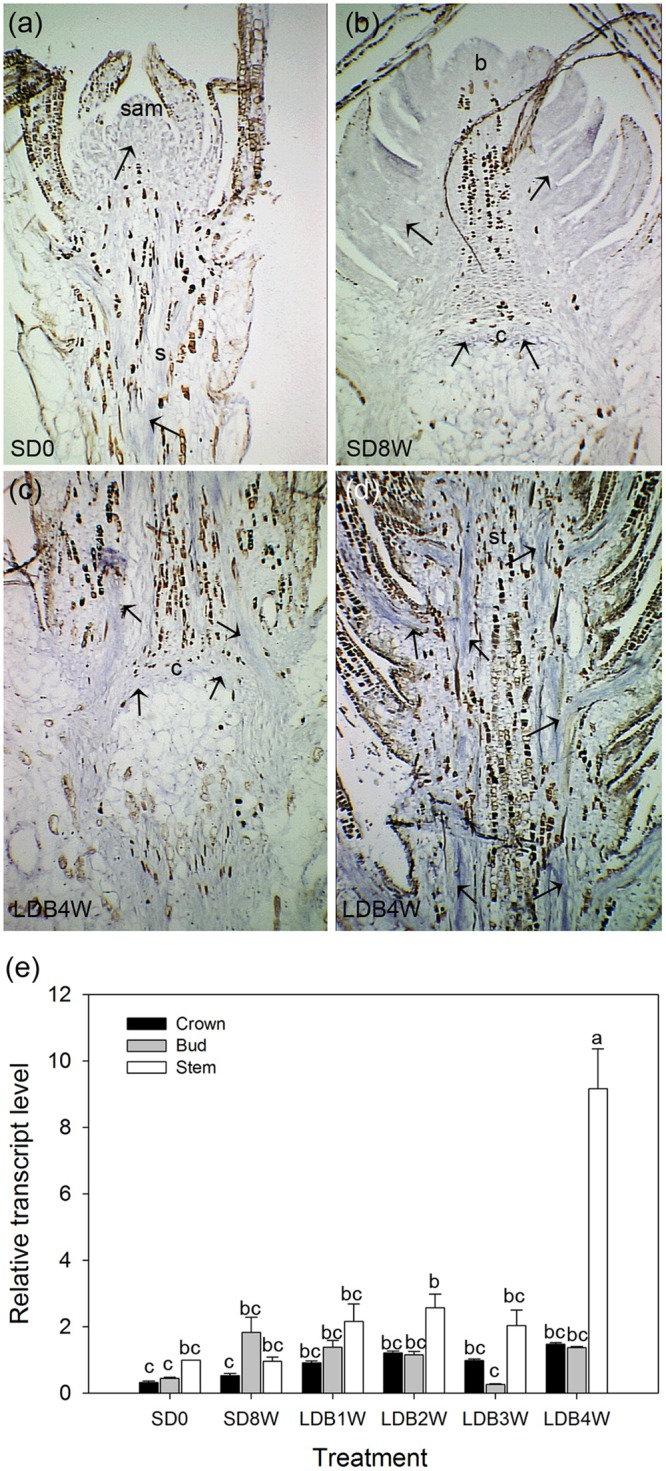
RNA *in situ* hybridization and transcript levels of an aquaporin gene in shoot tips, terminal buds, and breaking buds of Norway spruce. Arrows indicate areas of visible aquaporin expression (bluish color). **(a)** Longitudinal section of LD-grown shoot (LD = 0 short days (0SD). **(b)** Terminal bud after 8 weeks of SD treatment (SD8W). **(c)** Breaking bud with focus on crown region 4 weeks after re-transfer to LD following SD8W (LDB4W). **(d)** Elongating new stem tissues above the crown region in breaking bud at LDB4W. sam, shoot apical meristem; b, bud; c, crown; s, stem tissue; st, elongating stem in a breaking bud. Scale bars: 310 μm. **(e)** Transcript level of an aquaporin gene in different parts (bud; ca 2–3 mm, crown; ca 1 mm, stem beneath crown; ca 5 mm) below the crown of dissected shoot tips of LD day-grown plants, at SD8W and 1, 2, 3, and 4 weeks after re-transfer to LD following SD8W. For the LD (SD0)-grown plants 2–3 mm of the shoot tip (containing sam), 1 mm and 5 mm stem tissue beneath this were harvested for comparison. The values are mean ± SE of three samples consisting of pooled tissue from 8 to 10 plants for bud and stem and from 15 to 20 plants per treatment for crown. Different letters indicate significant differences between treatments and plant parts.

Transcript levels of an aquaporin gene were assessed by *in situ* localization and qPCR. In LD-grown plants, even distribution of relatively low levels of aquaporin transcript was observed in the shoot tip and stem tissue (**Figure 10a**). Plants exposed to SD for 8 weeks showed relatively low presence of aquaporin transcript in the terminal bud and crown (**Figure 10b**), which remained low 4 weeks after re-transfer to LD following 8 weeks of SD (**Figures 10c,d**). However, the qPCR results showed significantly higher aquaporin transcript level in stem tissue 4 weeks after re-transfer to LD, with higher levels (more than four-fold) compared to the other time points (**Figure 10e**).

## Discussion

In Norway spruce and a range of species of the pine family, the cell plate of thick-walled cells, denoted the crown, formed at the base of the buds (**Figures 1**, **2**) have been suggested to function as a barrier preventing freezing of the leaf primordia in the bud (Sakai, 1979; MacDonald and Owens, 1993). In this study, by addressing biochemical and anatomical characteristics of the crown region, we tested whether the crown region is an important SD-induced barrier inducing dehydration of the developing winter buds by preventing water influx.

The composition of the pectin matrix of the cell walls and particularly the degree of methyl-esterification of HG is a main determinant of the porosity of cell walls, and thus the movement of water molecules in the apoplast (Baron-Epel et al., 1988; Wisniewski et al., 1991b; Rajashekar and Lafta, 1996). Extensive binding of JIM7 antibodies against methyl-esterified HG and no detectable JIM5 epitopes of de-methyl-esterified HG in the cell walls of shoot tips under LD (**Figures 3**, **4**), is consistent with synthesis of HG pectin as the highly methyl-esterified form in shoot tips of growing plants as shown, e.g., in mustard (*Sinapis alba*) (Knox et al., 1990; Sobry et al., 2005; Xu et al., 2011). The opposite situation 3 and 8 weeks after transfer to SD, with increased and reduced JIM5 and JIM7 epitope abundance, respectively, in the crown region, strongly indicates a shift toward higher degree of de-methyl-esterification in response to SD. Furthermore, reversal (in the crown-region) toward the original LD situation 3 weeks after re-transfer to LD following 8 weeks in SD, supports photoperiod as an important determinant of the HG methyl-esterification status. Unlike the immunofluorescence experiment (**Figures 3f,g**), there was no significant increase in the JIM7 epitope 3 weeks after the re-transfer to LD in the IEM experiment (**Figures 4h,i**). The immunolabeling results were supported by mid-IR spectroscopy analyses demonstrating a shift toward higher wave numbers characteristic of higher degree of de-methyl-esterified HG in the crown region under SD as compared to LD (**Figure 6**). This resembles the situation in dormant floral buds and xylem in peach where more abundant de-methyl-esterified HG (JIM5 epitopes) was observed during the winter than in summer (Wisniewski and Davis, 1995).

Removal of methyl-groups from HG by PME enzymes allows binding of Ca^2+^ ions to the carboxylate ion (Grant et al., 1973; Knox et al., 1990; Braccini and Perez, 2001; Ralet et al., 2001; Willats et al., 2001; Xu et al., 2011). Thus, high and low abundance of Ca^2+^ ions in the cell walls and middle lamella of the crown region after 8 weeks under SD and 3 weeks after the re-transfer to LD, respectively (**Figure 5**), is consistent with the high and low amount of de-methyl-esterified HG in these treatments. Binding of Ca^2+^ ions results in crosslinking with formation of an orderly pectic gel with reduced pore size of the pectin cell wall matrix, which in turn limits movement of water molecules in the apoplast (Baron-Epel et al., 1988; Wisniewski et al., 1991a; Rajashekar and Lafta, 1996). According to this, the higher abundance of de-methyl-esterified HG and Ca^2+^ binding in the cell walls of the crown region at the base of the buds under SD is consistent with reduced pore size of the pectin matrix in this region. Thus, similar to what was suggested for dormant flower buds in peach (which do not have a crown structure) (Wisniewski and Davis, 1995), under SD the crown cells may contribute to limitation of the water flow into the buds.

Higher absorbance bands in the amide regions of proteins in the cell walls of the crown region under SD than after re-transfer to LD, might suggest an effect of photoperiod also on cell wall proteins. Such a change in the content of cell wall proteins is consistent with important functions in mechanical strengthening during development and stress through cross-linking and association with cell wall carbohydrates such as pectin and hemicelluloses (Keller, 1993; Tan et al., 2013; Tenhaken, 2014).

Our results indicate blocking of the plasmodesmata in the crown region with callose under SD and re-opening after re-transfer to LD (**Figure 7**). Although angiosperm trees do not develop a crown structure at the bud base, the situation in Norway spruce resembles that of birch (*Betula pubescens*) where callose-containing sphincters appear at plasmodesmata in the shoot apical meristem under SD, limiting symplastic transport (Rinne et al., 2001).

Lignin staining experiments revealed lack of xylem continuity across the crown into the SD-induced buds (**Figure 8b**). This is similar to the observation that dormant floral buds of *Prunus* lack xylem vessel elements and xylem continuity, which was suggested to be related to deep supercooling and avoidance of freezing injury (Ashworth, 1984). The dis-continued xylem connections suggests limitation of the water transport between the bud and the subtending shoot under SD. Indeed, the fluorescein and laurdan uptake experiments indicate no uptake of water into the buds under SD (**Figures 8f,g,j**). In contrast, consistent with the observed re-establishment of the xylem connections after re-transfer to LD (**Figures 8c,d**), uptake then occurred (**Figures 8h,k**). Thus, in addition to modification of the cell wall HG pectin and plasmodesmata blocking, discontinuity of the xylem at the base of the terminal bud is apparently an important structural mechanisms ensuring a dehydrated state of the apical bud and thus prevention of freezing injury in winter buds.

In agreement with the lack of fluorescein and laurdan uptake into buds, the water content in shoot tips decreased under SD and the situation was reversed after re-transfer to LD (**Figure 9**). These observations are consistent with the previous notion that the velocity of water transport in twigs and buds of Norway spruce trees is slow in the winter, and increase to a maximum level in spring before bud burst (de Faye et al., 2000). Furthermore, magnetic resonance imaging (MRI) results for apple trees revealed occurrence of free water only when the chilling requirement for dormancy alleviation was satisfied but not during deep dormancy (Liu et al., 1993). Similar results were reported for blueberries and grapes (Rowland et al., 1992; Gardea et al., 1994). Also, dormant flower buds of peach trees were shown to contain less water than flushing buds in spring (Yooyongwech et al., 2008).

Considering that aquaporin pores are the main sites for water transport across membranes, and that aquaporin transcript levels vary through the dormancy cycle in buds of peach trees (Yooyongwech et al., 2008, 2009), we addressed transcript localization and levels of an aquaporin gene in shoot tips and buds in Norway spruce. The substantial upregulation (about four-fold) in the stem tissues after re-transfer to LD following 8 weeks of SD (**Figure 10**), is consistent with the increased abundance of water upon bud break and resumption of growth. However, there were no significant difference in aquaporin transcript levels in the crown region and more studies of additional aquaporins are required to evaluate the significance of aquaporins in this respect. As yet, information on the role of aquaporins in Norway spruce is limited.

In summary, we here present evidence that SD-induced terminal bud formation in Norway spruce is associated with formation of multiple bud–shoot barriers limiting or preventing water transport into the bud. Among these are plasmodesmata blocking through callose deposition, and cell wall modification in cells of the crown region at the base of the bud, particularly increased de-methyl-esterification of the HG pectin, a processes known to decrease cell wall porosity and thus water permeability. Furthermore, discontinued xylem connections across the crown region adds to the restriction or blockage of the water transport into the bud. These changes are reversed after re-transfer to growth-inducing LD conditions. These findings are consistent with the suggested function of the crown structure in avoidance of freezing through action as water barrier (Sakai, 1979).

## Author Contributions

YL and JO devised and participated in all aspects of the studies including designing of the experiments, statistical analyses and interpretation of the results. YL contributed to growing plants and harvested the materials, performed microscopy studies, and participated in the synchrotron mid-IR analysis. CK, RL, and XL did and interpreted the synchrotron mid-IR analysis. KT coordinated the synchrotron mid-IR analysis and discussed experiments. YL, CK, JO, and KT participated in writing the manuscript. All authors have read and approved the final manuscript.

## Conflict of Interest Statement

The authors declare that the research was conducted in the absence of any commercial or financial relationships that could be construed as a potential conflict of interest.

## Footnotes

1 http://www.skogfroverket.no/
